# The effect of diode laser with PRP and pelvic floor chair for treatment of stress urinary incontinence in women

**DOI:** 10.6026/973206300212776

**Published:** 2025-08-31

**Authors:** Minie Anand, Winie Gautam, Garima Srivastava, Manjari Sinha, Nikhil Anand

**Affiliations:** 1 Department of Obstetrics & Gynecology, Madhubani Medical College, Madhubani, Bihar, India; 2 Consultant Pathologist, AMA Diagnostic Centre Pvt. Ltd., Lucknow, Uttar Pradesh, India; 3 Department of Obstetrics & Gynecology, Director & Sr Consultant at Orange Klinic, Gurgaon, Haryana, India; 4 Department of Orthopaedics, Bangalore Medical College & Research Institute, Bangalore, Karnataka, India

**Keywords:** Stress urinary incontinence, diode laser, platelet-rich plasma, pelvic floor chair, ICIQ-UI SF, FSFI, women's health

## Abstract

The stress urinary incontinence (SUI) is a wide-spread disorder in the female population and usually occurs due to the insufficient pelvic support structures. Therefore, it is of interest to assess the efficacy of diode laser therapy + platelet-rich plasma (PRP) compare with the diode laser with pelvic floor chair therapy among 50 women of 35-50 years. Both groups improved deeply in the responses on urinary symptoms, with more decline in ICIQ-UI SF scores recorded in the PRP group. However, the sexual function was better enhanced in the pelvic floor chair group. It is advocated that a specific therapy choice should be made depending on the profile of the symptoms.

## Background:

Stress urinary incontinence (SUI) is a common female urological disorder, which is distinguished by the uncontrolled passage of urine when subjects engage in activities that raise the abdominal pressure, i.e., coughing, sneezing, or exercising [1]. It explains the most prevalent type of urinary incontinence in women, which poses a serious impact on the quality of life, daily activity and psychological health [2]. The main causes of SUI are the loss of strength of the pelvic floor muscles and the connective tissue which holds the bladder and the urethra in place or incompetence of the urethral sphincter mechanism [3]. The causes of SUI development are related to vaginal birth, having passed the state of menopause, aging, obesity/overweight situation, previous pelvic surgery and long-term illness including any state of increased intra-abdominal pressure [4, 5]. The standard conservative treatment we have of SUI is behavioral changes, pelvic floor muscle training, biofeedback and pharmacological treatments. Surgery may be required in the form of mid-urethral slings or colposuspension though, in worse scenarios, raising the ratio may indicate surgical urethropexy [6]. The latest type of interest is on non-invasive and minimally invasive options like intravaginal laser therapy and platelet-rich plasma (PRP) injections that rely on treating the root cause of the condition (tissue regeneration and collagen remodeling) [7, 8]. Photothermal stimulation exerts a benefit in the form of neocollagenesis, angiogenesis and tightening of the vaginal wall and periurethral tissues via diode laser therapy that has shown a prospective benefit in the whole idea of urethral support and continence mechanisms [9].

Conversely, PRP composed of the patient autologous blood has a high amount of growth factors inducing fibroblastoid action to repair tissue, which too can help to restore the sphincteric functionality [10, 11]. Moreover, because of the use of high-intensity focused electromagnetic (HIFEM) stimulation pelvic floor chair results in a supramaximal contraction of pelvic floor muscles, which results in frankly increased pelvic floor muscle strength and control of continence [12, 13]. Although PRP have been extensively utilized across various medical specialties, including orthopedics, urology, ophthalmology, aesthetic medicine and dermatology, its application in urogynecology remains relatively new and emerging [14]. Even though the individual benefits of these modalities have been demonstrated, comparative evidence regarding their combined use is limited. Therefore, it is of interest to evaluate and compare the therapeutic efficacy of diode laser combined with PRP versus diode laser combined with pelvic floor chair in women with SUI.

## Materials and Methods:

This prospective observational study was conducted at Sun Hospital and included 50 female participants between the ages of 35 and 50 years diagnosed with stress urinary incontinence (SUI). The objective was to compare the therapeutic outcomes of diode laser therapy combined with platelet-rich plasma (PRP) and diode laser therapy combined with pelvic floor chair sessions. Participants were divided into two groups of 25 each. The first group received monthly sessions of vaginal diode laser therapy in conjunction with twice-weekly pelvic floor chair therapy for a duration of six weeks. The second group underwent the same diode laser protocol, but instead of chair therapy, they received paraurethral PRP injections once a month for three months. All participants were followed up for six months, with assessments at baseline, one month and six months post-treatment.

## Inclusion and Exclusion criteria:

Women included in the study had clinically diagnosed SUI without concurrent genital tract infections or recent pelvic surgeries (within the past three months). Exclusion criteria included pregnancy, presence of metallic implants, coagulation disorders, malignancies, or any condition that could interfere with treatment safety or outcome interpretation.

## Intervention protocols:

## Diode laser therapy:

All participants underwent diode vaginal laser therapy using a 1470 nm wavelength device set to 17 watts in pulsed mode (5 Hz frequency, 50 ms pulse duration). Each session lasted approximately 20 minutes and was administered monthly for three sessions.

## Pelvic floor chair:

In the group receiving pelvic floor chair therapy, sessions were scheduled twice weekly for six weeks, excluding laser treatment days. The chair delivered high-intensity focused electromagnetic (HIFEM) stimulation up to 2.5 Tesla, with the energy reaching a depth of 10 cm to induce supramaximal pelvic floor muscle contractions. Each session lasted 28 minutes.

## Platelet-Rich Plasma (PRP) Injections:

PRP was prepared using a standardized dual-spin centrifugation method. A total of 45 ml of autologous blood was collected into three sodium citrate-containing tubes. The initial centrifugation was performed at 1500 rpm for 15 minutes, followed by a second spin at 3500 rpm for 10 minutes. The resulting platelet-rich fraction (~1.5 ml per tube) was extracted and injected into the anterior vaginal wall near the mid-urethra, approximately 1 cm below the urethral meatus, with a depth of about 1.5 cm. The total volume injected was 5 ml per session (2 ml beneath the urethra and 1.5 ml on each side), administered monthly for three months without anesthesia.

## Outcome measures:

Symptom severity and treatment response were evaluated using the International Consultation on Incontinence Questionnaire - Urinary Incontinence Short Form (ICIQ-UI SF), Urogenital Distress Inventory (UDI-6), Incontinence Impact Questionnaire (IIQ-7) and the pad test. Additionally, changes in sexual function were assessed using the Female Sexual Function Index (FSFI). These assessments were carried out at baseline, 1 month and 6 months after treatment initiation. All collected data were analyzed statistically, with significance set at p<0.001 for pre- and post-treatment comparisons.

## Results:

This study evaluated the comparative effectiveness of diode laser combined with platelet-rich plasma (PRP) versus diode laser combined with pelvic floor chair therapy in women with stress urinary incontinence (SUI), based on symptom improvement and functional scores over a follow-up period of six months. Both groups exhibited significant improvements in urinary symptoms as assessed by the International Consultation on Incontinence Questionnaire - Urinary Incontinence Short Form (ICIQ-UI SF). However, the group treated with diode laser and PRP demonstrated more pronounced improvement across all symptom parameters compared to the group receiving diode laser with pelvic floor chair therapy. As shown in Table 1 (see PDF), the mean total ICIQ-UI SF score in the laser + PRP group decreased from 14.1 ± 2.8 at baseline to 5.6 ± 3.2 post-treatment (p<0.001), whereas the laser + pelvic floor chair group showed a reduction from 14.6 ± 3.7 to 7.2 ± 2.9 (p<0.001). Additionally, improvements were noted in urgency, frequency, incontinence episodes and the use of absorbent pads (Table 1 - see PDF). Severity of SUI was classified into mild, moderate and severe categories. Notably, 60% of patients in the laser + PRP group showed clinical improvement, compared to 48% in the laser + pelvic chair group. As detailed in Table 2 (see PDF), complete cure (defined as shift to 'no incontinence') was observed in 13 patients in the PRP group and 9 patients in the chair group. Sexual function, assessed using the Female Sexual Function Index (FSFI), improved more significantly in the diode laser + pelvic floor chair group than in the PRP group. As per Table 3 (see PDF), the laser + chair group experienced a notable increase in FSFI total score from 17.4 ± 4.2 to 25.9 ±5.0 (p<0.001), while the PRP group saw negligible change (17.9 ± 10.2 to 17.9 ± 10.4; p<0.001). All individual domains-lubrication, pain, desire, arousal, orgasm and satisfaction-showed greater improvement in the chair group.

[1] Urinary symptoms improved in both groups, with greater improvement seen in the diode laser + PRP group (Table 1 - see PDF).

[2] SUI severity grading showed a higher rate of clinical improvement in the PRP group compared to the pelvic chair group (Table 2 - see PDF).

[3] Sexual function showed substantial enhancement in the pelvic floor chair group, while remaining relatively unchanged in the PRP group (Table 3 - see PDF).

## Discussion:

Stress urinary incontinence (SUI) is a big problem of urological and gynecological nature, mainly associated with middle-aged female population. It has implications on quality of life, mental health and sexual health. The cause of the condition is the weakening process of the urethral sphincter and supportive structures of the bladder and urethral, which may be affected by various issues, including childbirth, the decline of hormones, age and surgery of the pelvis [15]. Over the past few years, there has been the introduction of non-surgical modalities which are promising alternatives to invasive treatment especially to women who desire a minimally invasive procedure with reduced complications [16]. This examination was set out to determine the similarity between two combined-therapeutic systems diode laser with platelet-rich plasma (PRP) and diode laser with pelvic floor chair therapy in terms of their efficacy. Diode laser has a therapeutic effect through the photothermal energy that causes collagen remodeling and the enhancement of tissue elasticity and mucosal support inside the vaginal and perinethral areas [5, 6]. In turn, PRP provides high-intensity pool of growth factors such as platelet derived growth factor (PDGF), transforming growth factor-beta (TGF-beta) and vascular endothelial growth factor (VEGF) that were associated to tissue repair and angiogenesis [17]. On the other hand, pelvic floor chair technology provides high-intensity focused electromagnetic (HIFEM) waves so as to produce supramaximal contractions of pelvic muscles, leading to increasing urethral support and control over urinary leakage [18]. This study shows that both of the combinations of treatment have result which is statistically significant on the ICIQ-UI SF report after a 6-month follow-up. Nevertheless, a decrease in urgency, frequency, the number of episodes of incontinence and absorbent pad usage was also more noticeable in the diode laser + PRP group than in the pelvic floor chair group (Table 1 - see PDF). These results match those of the previous studies that displayed PRP injections as being especially beneficial in treating mild-moderate SUI, with exhibited tissue-generating effects and volumetric urethra support [11, 12]. Furthermore, a higher proportion of patients in the PRP group achieved complete resolution or improvement in SUI severity compared to the pelvic chair group (60% vs. 48%) (Table 2 - see PDF). Previous studies have shown that injectable therapies such as PRP can result in improved urethral coaptation and mid-urethral function, thus offering promising results in SUI management [13]. In contrast, HIFEM-based therapies, while non-invasive and well tolerated, might require ongoing sessions for sustained effect [19]. Interestingly, when analyzing the FSFI scores, women treated with the pelvic floor chair exhibited significantly greater improvements in sexual function parameters-including lubrication, arousal, orgasm and overall satisfaction-compared to those receiving PRP (Table 3 - see PDF). This aligns with earlier reports indicating that HIFEM therapy may enhance neuromuscular re-education and pelvic floor tone, which could contribute to better sexual responsiveness and reduced dyspareunia [20, 21]. The PRP group, while showing improved urinary symptoms, did not exhibit significant enhancement in sexual domains, possibly due to the localized nature of the injection and absence of muscular stimulation [22]. Despite encouraging outcomes, this study has limitations. The absence of a control group restricts the ability to draw definitive conclusions. Additionally, the relatively small sample size may limit generalizability. Self-reported outcome measures, though validated, are subject to recall and reporting biases. These findings should, therefore, be interpreted cautiously and verified through randomized controlled trials involving larger and more diverse cohorts [23, 24]. Overall, this study supports the clinical value of combining diode laser therapy with either PRP or pelvic floor chair therapy for treating SUI. Diode laser with PRP appears more effective for improving urinary symptoms, while diode laser with pelvic floor chair therapy shows superior benefits in sexual function domains. The choice of therapy should be tailored to individual patient needs and presenting symptoms.

## Conclusion:

Diode laser therapy combined with PRP demonstrated superior improvement in urinary symptoms compared to its combination with pelvic floor chair therapy. However, pelvic chair therapy showed greater enhancement in sexual function. Individualized treatment selection is essential based on patient-specific needs and symptom profile.
